# Cytotoxic Aβ Protofilaments Are Generated in the Process of Aβ Fibril Disaggregation

**DOI:** 10.3390/ijms222312780

**Published:** 2021-11-26

**Authors:** Toshisuke Kaku, Kaori Tsukakoshi, Kazunori Ikebukuro

**Affiliations:** Department of Biotechnology and Life Science, Tokyo University of Agriculture and Technology, 2-24-16, Naka-cho, Koganei, Tokyo 184-8588, Japan; t.kaku0801@gmail.com

**Keywords:** Alzheimer’s disease, disaggregation, amyloid β, protofilaments, EGCG, dopamine

## Abstract

Significant research on Alzheimer’s disease (AD) has demonstrated that amyloid β (Aβ) oligomers are toxic molecules against neural cells. Thus, determining the generation mechanism of toxic Aβ oligomers is crucial for understanding AD pathogenesis. Aβ fibrils were reported to be disaggregated by treatment with small compounds, such as epigallocatechin gallate (EGCG) and dopamine (DA), and a loss of fibril shape and decrease in cytotoxicity were observed. However, the characteristics of intermediate products during the fibril disaggregation process are poorly understood. In this study, we found that cytotoxic Aβ aggregates are generated during a moderate disaggregation process of Aβ fibrils. A cytotoxicity assay revealed that Aβ fibrils incubated with a low concentration of EGCG and DA showed higher cytotoxicity than Aβ fibrils alone. Atomic force microscopy imaging and circular dichroism spectrometry showed that short and narrow protofilaments, which were highly stable in the β-sheet structure, were abundant in these moderately disaggregated samples. These results indicate that toxic Aβ protofilaments are generated during disaggregation from amyloid fibrils, suggesting that disaggregation of Aβ fibrils by small compounds may be one of the possible mechanisms for the generation of toxic Aβ aggregates in the brain.

## 1. Introduction

Alzheimer’s disease (AD) is a major cause of dementia, and many researchers are attempting to develop diagnostic methods and drastic treatments. One of the major factors involved in the onset of AD is the amyloid β (Aβ) peptide, as determined from the observation of amyloid plaques composed of Aβ in the brains of patients with AD [1]. Moreover, the oligomeric state of Aβ is large in quantity in patients with AD [2], provokes neuronal cell death and memory deficit [3,4], and is correlated with cognitive impairment [5], suggesting that it is a primary toxic agent [6]. Thus, revealing the mechanism of generation of toxic Aβ oligomers is crucial for understanding AD pathogenesis.

To understand the buildup of Aβ oligomers, the aggregation process of Aβ has mostly been investigated. In this process, intrinsically disordered Aβ monomers spontaneously aggregate to form mature insoluble amyloid fibrils, and various types of Aβ oligomers are generated as intermediate products [6]. In aggregation processes, small and globular oligomers grow into protofilaments, in which several Aβ monomers are stacked to form a typical cross-β-sheet structure. Several protofilaments are twisted to form linear or curly chain-like protofibrils, and then elongated to form ordered mature fibrils [7,8,9]. High-molecular-weight (HMW) oligomers (protofilaments and protofibrils) show higher cytotoxicity than monomers and mature fibrils, because the β-sheet-rich structure of the Aβ oligomers is likely to interact with the cell membrane and form channel-like pores to disrupt the homeostasis of neural cells [10]. Thus, revealing the generation process of toxic intermediate products is important for therapeutic targeting.

As a dynamic process with the structural change of Aβ, in addition to the aggregation process, the disaggregation process of Aβ fibrils was discovered. Many reports showed that amyloid fibrils can be disaggregated by treatment with chemical compounds, such as polyphenol [11,12,13,14]. In silico studies showed that the aromatic and hydroxy groups of chemical compounds mainly interact with the hydrophobic core and salt bridge of Aβ, respectively, leading to fibril dissociation [15,16,17]. In vitro studies showed the loss of the fibril structure by atomic force microscopy (AFM) or transmission electron microscopy imaging, and lower cytotoxicity of Aβ after full disaggregation [11]. However, the cytotoxicity and structural features of intermediate Aβ products during the Aβ fibril disaggregation process are poorly understood. Considering the disaggregation process, toxic HMW Aβ aggregates, such as protofilaments and protofibrils, are highly likely to be generated in the initial phase of fibril disaggregation, which is a disassembly of mature amyloid fibrils consisting of Aβ.

Based on the expectation that the disaggregation of Aβ fibrils may cause the generation of toxic Aβ oligomers, we investigated whether toxic Aβ oligomers, such as protofilaments and protofibrils, could be generated during the process of Aβ fibril disaggregation. In this study, we first tested the most popular disaggregating chemical compound: the green tea polyphenol epigallocatechin gallate (EGCG) [11]. Thioflavin T (ThT) was utilized to monitor fibril disaggregation and determine the optimal conditions for collecting moderately disaggregated Aβ species. A cell viability assay, AFM imaging, and circular dichroism (CD) spectrometry measurements were performed to determine the cytotoxicity and structure of moderately disaggregated Aβ aggregates. We also tested dopamine (DA), a neurotransmitter found in the brain, as a small compound that disassembles amyloid fibril structures.

## 2. Results

### 2.1. Mild Disaggregation of Aβ Fibrils by EGCG and DA to Produce Cytotoxic Aβ Aggregates

**Figure 1:**
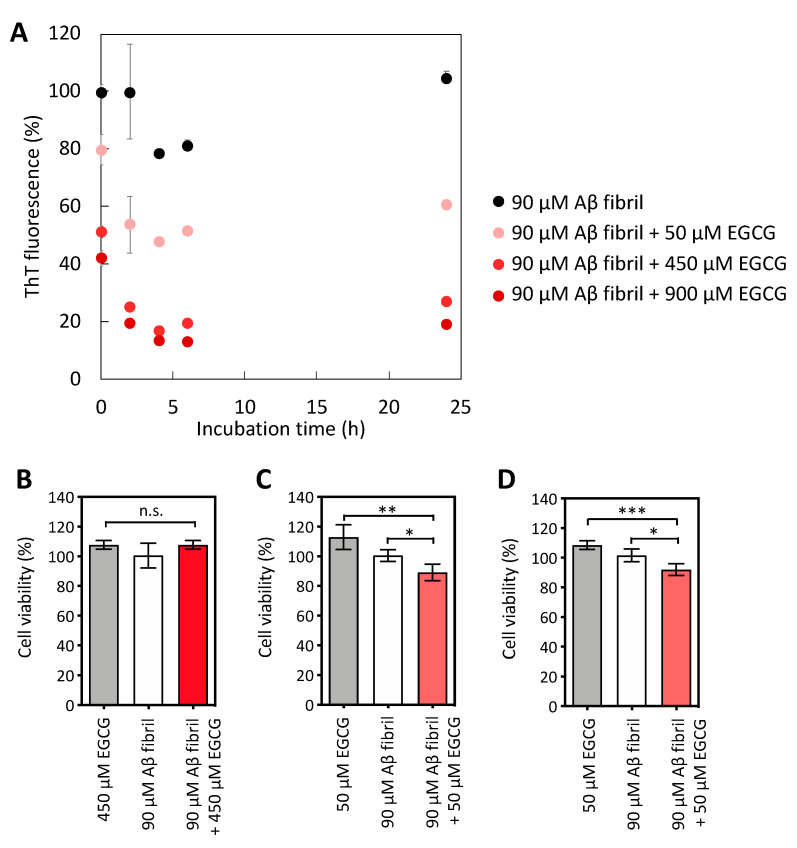
Concentration- and time-dependent effects of epigallocatechin gallate (EGCG) on the amyloid β (Aβ) fibril disaggregation and cytotoxicity. (**A**) Normalized Thioflavin T (ThT) fluorescence intensity during fibril disaggregation in the presence of different molar ratios of EGCG. The fluorescence intensity of Aβ fibrils without compounds at 0 h is indicated as 100%. The mean ± standard deviation (SD) is shown on the graph (N = 3). The cytotoxicity of Aβ samples disaggregated in (**B**) 450 µM of EGCG for 24 h, (**C**) 50 µM of EGCG for 6 h, and (**D**) 50 µM of EGCG for 24 h was investigated using the MTS assay. Additionally, 100% cell viability represents the cell viability when incubated with the solvent control: diluted dimethyl sulfoxide with phosphate-buffered saline. The cytotoxicity data are shown as the mean ± SD on the graph (N = 4). Statistical analysis was performed using a two-way analysis of variance (ANOVA) with Tukey’s multiple comparison test: * *p* < 0.05, ** *p* < 0.01, and *** *p* < 0.001.

Different concentrations (50, 450, and 900 µM) of EGCG were added to 90 µM of Aβ fibrils, and the mixture was incubated for several periods to determine the preparation conditions to obtain Aβ samples rich in toxic Aβ oligomers. Figure 1A shows the change in relative ThT fluorescence intensity during the disaggregation assay of Aβ fibrils at 37 °C. Under the EGCG mixing conditions, the ThT fluorescence intensity decreased in a compound concentration- and time-dependent manner compared to the Aβ fibrils alone (Figure 1A). After the addition of 450 or 900 µM of EGCG to the Aβ fibrils, the ThT fluorescence derived from mature amyloid fibrils immediately decreased from 0 to 4 h, which indicated a rapid loss of the amyloid fibril structure during 4 h of incubation. The ThT fluorescence intensity from the Aβ fibrils mixed with 450 or 900 µM of EGCG showed an 80% or 87% reduction, respectively, after 6 h of incubation (Figure 1A; bright red circle and dark red circle). Therefore, as reported previously by others [11], the Aβ fibrils were disrupted by incubation with EGCG under high molar equivalent conditions (Aβ:EGCG = 1:5 and 1:10). Since the ThT fluorescence values measured at 4 and 6 h were similar, we considered that the disaggregation of the Aβ fibrils was complete. Therefore, we did not measure the fluorescence values from 7 to 23 h, but we measured the fluorescence at 24 h to confirm that the value did not change.

To prepare intermediate species in the Aβ fibril disaggregation process, we also tested the incubation of EGCG with Aβ fibrils under low molar equivalent conditions (Aβ:EGCG = 1:0.56). The Aβ fibrils incubated with 50 µM of EGCG also showed a time-dependent decrease in ThT fluorescence for the first 4 h, and 50% of ThT fluorescence signals remained after 6 h of incubation (Figure 1A; pink circle). Compared to the ThT fluorescence resulting from the addition of 450 or 900 µM of EGCG, the intensity at 50 µM was significantly higher (Figure 1A). Because a decreased signal of fluorescent dyes in the investigation of amyloid formation, such as ThT and Congo red, indicates the refolding of the amyloid fibril structure to form non-fibrillar structures via the binding or interaction of EGCG with the fibril structure [11,18], a comparison of the results with 50, 450, and 900 µM of EGCG suggested that Aβ fibrils could be partially disaggregated under low molar equivalent conditions. Therefore, we hypothesized that the mild disaggregation of Aβ fibrils with EGCG could produce an increase in HMW Aβ aggregates, such as Aβ protofilaments.

We analyzed the cytotoxicity of the Aβ samples treated with 50 µM of EGCG for neuroblastoma SH-SY5Y cells using the MTS assay and compared the results with those of the disaggregated Aβ with 450 µM of EGCG. First, we confirmed the absence of cytotoxicity in the Aβ fibril sample treated with 450 µM of EGCG for 24 h (Figure 1B). Therefore, as reported previously by others [19] and as suggested by the ThT fluorescence measurements (Figure 1A; bright red circles), the Aβ fibril sample mixed with EGCG at a molar ratio of 1:5 for 24 h was fully disaggregated, resulting in no cytotoxicity against SH-SY5Y cells. In contrast, the addition of the Aβ sample preincubated with 50 µM of EGCG for 6 and 24 h resulted in a significant decrease in cell viability compared to the Aβ sample without EGCG (Figure 1C,D). This cytotoxicity was not observed in the Aβ sample collected immediately after mixing it with EGCG (data not shown), suggesting that the cytotoxicity was caused by the mild disaggregation of Aβ by EGCG.

**Figure 2:**
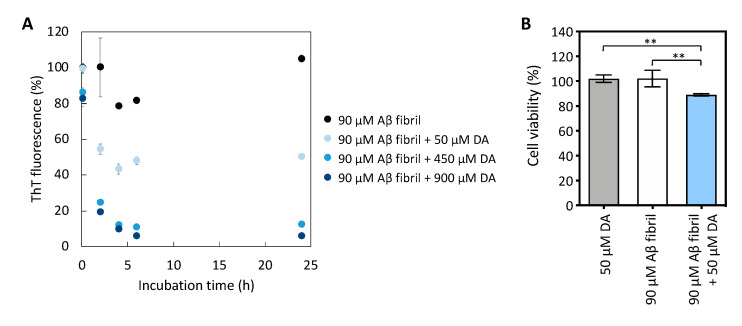
(**A**) Normalized Thioflavin T (ThT) fluorescence intensity during fibril disaggregation in the presence of different molar ratios of dopamine (DA). The fluorescence intensity of amyloid β (Aβ) fibrils without compounds at 0 h is indicated as 100%. The mean ± standard deviation (SD) is shown on the graph (N = 3). The fluorescence data of 90 µM Aβ fibrils alone are the same as those in Figure 1A because the experiments using epigallocatechin gallate and DA were performed simultaneously. (**B**) The cytotoxicity of Aβ samples disaggregated in 50 µM of DA for 5 h was evaluated. The cytotoxicity data are shown as the mean ± SD on the graph (N = 4). Additionally, 100% of cell viability represents the cell viability when incubated with the solvent control: diluted dimethyl sulfoxide with phosphate-buffered saline. Statistical analysis was performed using two-way analysis of variance (ANOVA) with Tukey’s multiple comparison test: ** *p* < 0.01.

If toxic Aβ oligomers can be produced by the disaggregation of Aβ fibrils, then compounds with disaggregating effects present in the brain may be involved in the generation of toxic Aβ oligomers related to the onset of AD. Therefore, we next tested the effect of DA, a neurotransmitter that has a disaggregation effect on Aβ fibrils [12]. We found that DA, as in the case of EGCG, promoted the disaggregation of Aβ fibrils in a concentration- and time-dependent manner (Figure 2A). Next, we examined the viability of SH-SY5Y cells incubated with disaggregated Aβ samples using 450 µM of DA. However, because a high concentration of DA alone caused cell death in SH-SY5Y cells (data not shown), as shown previously by Liu et al. [20], we could not determine whether full disaggregation of Aβ fibrils by DA abolished its cytotoxicity. Despite this, we found that 50 µM of DA was not toxic to SH-SY5Y cells (Figure 2B). Therefore, we determined that 50 µM of DA partially disaggregated Aβ samples. Similar to the results shown in Figure 1C, we observed that 90 µM Aβ samples incubated with 50 µM of DA under a low molar equivalent condition (Aβ:DA = 1:0.56) showed higher cytotoxicity than Aβ fibrils without the addition of DA (Figure 2B). These results imply that low concentrations of chemical compounds with disaggregation activity can moderately promote Aβ fibril disaggregation to produce toxic Aβ species.

### 2.2. AFM Imaging Analysis of Disaggregated Aβ Fibrils

**Figure 3:**
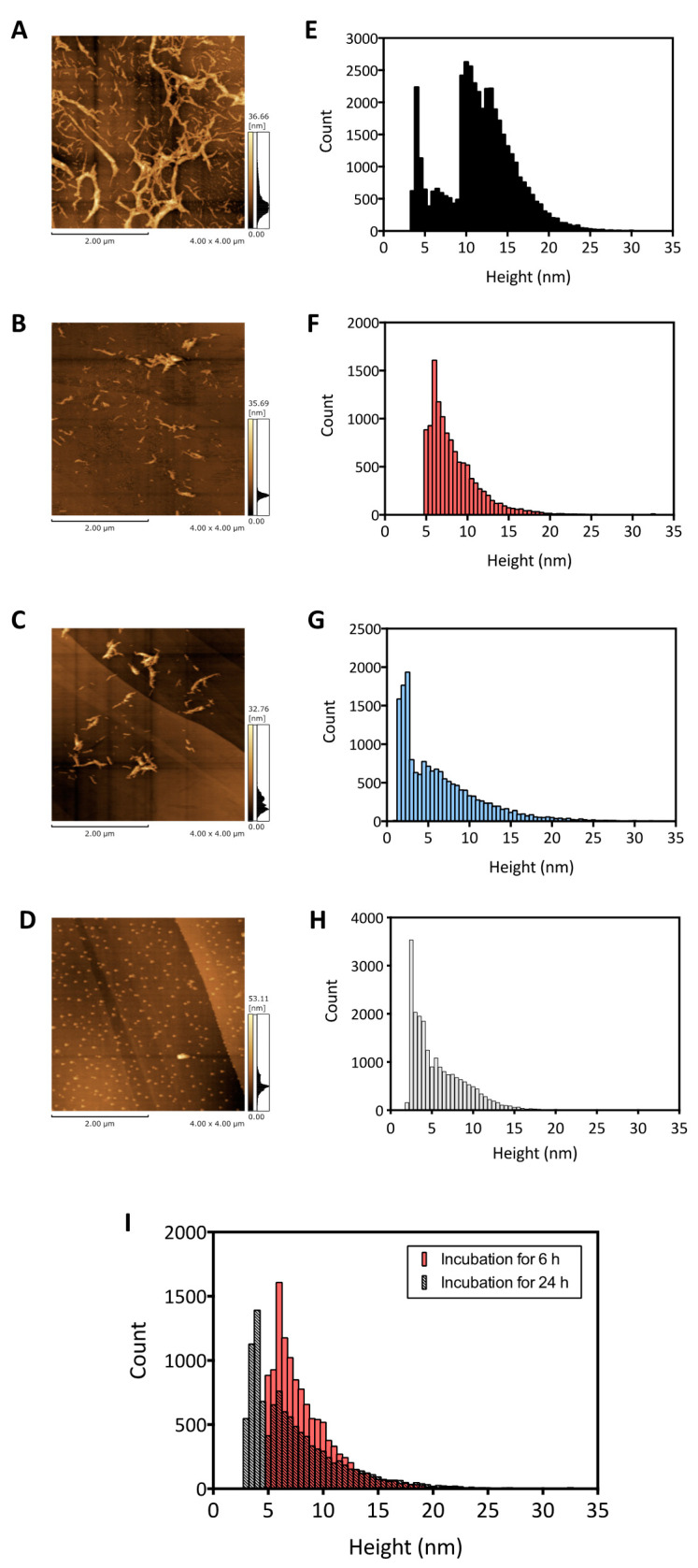
Representative atomic force microscopy images and histogram of the height distribution of untreated amyloid β (Aβ) fibrils (**A,E**), Aβ fibrils incubated for 6 h with epigallocatechin gallate (EGCG) (**B,F**), Aβ fibrils incubated for 6 h with dopamine (DA) (**C,G**), and Aβ before fibrillization (**D,H**); Aβ fibrils mixed with EGCG or DA at a molar ratio of 1:0.56 (**I**); time dependence of height distribution of Aβ incubated in the presence of EGCG for 6 h (pink bar, same data as (**F**)) and 24 h (black shaded bar).

For the structural characterization of the toxic Aβ aggregates, the morphology of the disaggregated Aβ was evaluated using AFM. After incubation for a minimum of 90 h at 22 °C, the Aβ became fibrillar aggregates with a length of more than 1 μm and a width of approximately 90 nm (Figure 3A), which is consistent with the results of the ThT analysis (Figure 1A and Figure 2A; black circle). In the AFM image of the Aβ fibrils mixed with EGCG and DA under a low molar equivalent condition (Aβ:EGCG or DA = 1:0.56), we observed shorter fibril-like structures, and not the intact Aβ fibrils (Figure 3A), with a length of less than 400 nm and a width of less than 50 nm (Figure 3B,C). The size of the disaggregated Aβ using 50 µM of EGCG or DA was larger than that of the Aβ before fibrillization (Figure 3D), indicating that the EGCG- or DA-treated Aβ was still aggregated.

We also quantitatively analyzed the height of the structures obtained from the AFM images. We investigated the distribution of the Aβ aggregate diameter. As shown in the histogram, the height of the untreated Aβ fibrils was mainly around 10–15 nm (Figure 3E). In contrast, the Aβ sample mixed with EGCG for 6 h showed more structures with heights of less than 5–10 nm (Figure 3F). In the sample mixed with DA for 6 h, there were many structures with a height of less than 5 nm, and the main peak in the histogram was at 2.5 nm in height (Figure 3G). We also characterized the height of the Aβ sample before fibrillization and confirmed that the peak of the histogram was at 2.5 nm (Figure 3H). It was previously reported that mature Aβ fibrils were formed by twisting several Aβ protofilaments, and the width of one of the Aβ molecules in the Aβ protofilament was approximately 2.5 nm [7,21]. From this information, we inferred that the Aβ aggregates with a fibrillary structure around 2.5 nm in height might correspond to one Aβ protofilament. Therefore, we expected the Aβ aggregates generated by the DA treatment to be Aβ protofilaments (Figure 3C,G). Additionally, according to the results of the cell toxicity experiments of the disaggregated Aβ samples (Figure 2B), the Aβ protofilaments generated by DA treatment may be causative aggregates, leading to a decrease in neuronal cell viability.

Incubation with EGCG for 6 h resulted in increased amounts of Aβ fibrillary structures of around 6 nm in height (Figure 3F). Since the height of one Aβ protofilament could be expected to be approximately 2.5 nm from previous reports [7,21], and according to the AFM images of the disaggregated Aβ and Aβ before fibrillization (Figure 3C,G,H), we expected that two Aβ protofilaments with a height of 2.5 nm each would be stacked, resulting in a bundle of Aβ aggregates with a height of approximately 6 nm. Interestingly, fibrillar Aβ structures consisting of two stacked Aβ protofilaments containing a cross-β-sheet structure were already found in the aggregation process from Aβ monomers to fibrils, and their structures were determined by cryo-electron microscopy [7,22,23]. In contrast to these previous observations, we considered that our results indicated that fibrillar Aβ composed of two Aβ protofilaments may be generated stably, even in the disaggregation process.

To determine whether EGCG could disaggregate an Aβ fibril to a single Aβ protofilament in the same manner as DA, we extended the incubation time with EGCG from 6 to 24 h. As a result, 24 h of incubation of EGCG with Aβ fibrils increased the amount of Aβ fibrillary structures around 3 nm in height (Figure 3I), as expected, suggesting that disaggregation from the Aβ aggregates, including two Aβ protofilaments to single Aβ protofilaments, was promoted by long-term incubation with EGCG. Because the Aβ fibrils that were treated with EGCG for 6 and 24 h (Figure 1C,D) were cytotoxic, we considered that EGCG could disaggregate Aβ fibrils to cytotoxic Aβ protofilaments, like DA.

### 2.3. Characterization of Secondary Structures of the Aβ Protofibrils Produced by Disaggregation Using EGCG and DA

**Figure 4:**
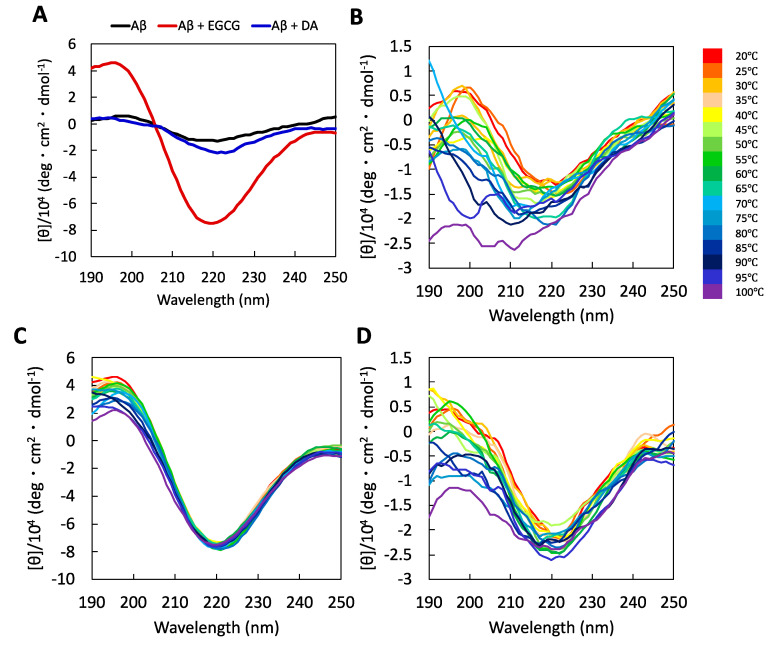
(**A**) Circular dichroism (CD) spectra of Aβ fibrils (black line). Amyloid β (Aβ) samples incubated with epigallocatechin gallate (EGCG; red line) or dopamine (DA; blue line) for 6 h were recorded at 25 °C. Aβ fibrils were mixed with EGCG or DA at a molar ratio of 1:0.56. Temperature-dependent CD spectra from 20–100 °C of Aβ fibrils alone (**B**) and in the presence of EGCG (**C**) or DA (**D**) were also recorded.

Aβ is intrinsically disordered in its monomeric state and gains a β-sheet structure during fibrillization. Given that the disaggregation process is the reverse phenomenon of the aggregation process, the β-sheet structure of amyloid fibrils was expected to be destabilized after disaggregation. In this regard, the secondary structure and thermal stability were analyzed using CD spectrometry. Aβ fibrils incubated with a 0.56 molar equivalent of EGCG or DA for 6 h remained to form a β-sheet structure according to a positive peak at around 195 nm and a negative peak at around 220 nm (Figure 4A). These results support the presence of Aβ protofilaments resulting from mild disaggregation by EGCG and DA. The EGCG-treated Aβ sample showed the strongest CD spectrum, with a peak at 220 nm, compared to the DA-treated Aβ sample. Because the AFM imaging study showed that the height of the EGCG-treated Aβ sample was higher than that of the DA-treated Aβ sample (Figure 3F,G), the structural difference may be the cause of the strongest CD intensity. Next, the thermal stability of Aβ was evaluated by measuring the CD spectra while changing the solution temperature. The spectra of the Aβ fibrils changed considerably above 70 °C, and the β-sheet-derived spectral peak at around 220 nm disappeared (Figure 4B). Surprisingly, the Aβ sample mixed with EGCG or DA showed a negative peak at around 220 nm even at temperatures above 70 °C (Figure 4C,D), which is different from the Aβ fibrils incubated under the same conditions in the absence of compounds (Figure 4B). Therefore, EGCG- and DA-treated Aβ protofilaments might have a higher stability in the β-sheet structure, which may be related to cytotoxicity. The reason for the high stability is still unknown, but a decrease in solvent exposure in the β-sheet region by EGCG and DA may contribute to the increased thermal stability [24].

## 3. Discussion

In this study, we revealed the generation of toxic Aβ protofilaments during Aβ fibril disaggregation. Disaggregation is a phenomenon in which the amyloid fibril structure collapses, and its morphology disappears. The incubation of Aβ fibrils with low molar equivalents of EGCG and DA resulted in moderately disaggregated Aβ and formed a fibril-like structure different from that of mature fibrils in length and height, corresponding to Aβ protofilaments. In addition, the evaluation of the secondary structure by CD spectrometry showed a higher stability of the β-sheet structure in these Aβ protofilaments, which may contribute to the higher cytotoxicity.

Recent works based on the oligomer hypothesis strongly facilitated investigation of the structure–toxicity relationship of Aβ oligomers. In the last 20 years, various toxic Aβ oligomers, such as Aβ dimers, trimers, and dodecamers, have been identified, supporting the oligomer hypothesis [6]. Regarding Aβ protofilaments, Stroud et al. demonstrated the presence of cytotoxic Aβ protofilaments with cross-β structures in vitro [25]. On the other hand, assisted by the discovery of Aβ protofilament structures [7,22,23], molecular dynamics simulation-based studies using the solved Aβ protofilament structure revealed the factors of its neurotoxicity, such as pore formation [26]. However, there are few reports on how Aβ protofilaments form; in general, toxic Aβ protofilaments are thought to be formed from Aβ monomers. In this paper, by detailed research on the disaggregation process of Aβ fibrils, we provide an answer to the question about the generation of toxic Aβ protofilaments and the direct evaluation of Aβ protofilament toxicity.

Because both EGCG and DA dissociated Aβ fibrils into Aβ protofilaments, small compounds with disaggregation effects, such as catechol derivatives and polyphenols (EGCG, DA, noradrenaline, etc.), could be involved in the generation of toxic Aβ protofilaments. In the present study, DA seemed to affect the dissociation of the Aβ fibrils more intensely than EGCG; the size of the dissociated Aβ fibrils after 6 h of incubation with DA (Figure 3G) was smaller than after 6 h of incubation with EGCG (Figure 3F). Considering that the decrease in the ThT fluorescence value from 0 to 6 h with the addition of 50 µM of DA (Figure 2A) was steeper than the decrease with the addition of 50 µM of EGCG (Figure 1A), the molecular mechanism for Aβ fibril disaggregation may be different between DA and EGCG. Recently, Wei and colleagues reported that both DA and EGCG disrupt the salt bridges between K28 and A42 by molecular dynamics (MD) simulations using the solved Aβ protofibril structure [17,27]. Additionally, MD simulations showed that DA bound to the hydrophobic site containing F4, L34, and V36 disrupts Aβ protofibrils, while EGCG breaks the H-bond between H6 and E11 of Aβ protofibrils [17,27]. Similar to our results, the MD simulation studies indicate that the effects of DA and EGCG are different. Therefore, the previous MD simulation and our study indicate that EGCG and DA might differ in their mechanism to disrupt Aβ fibrils, including the generation of toxic Aβ protofilaments.

Our final question concerns the possibility that Aβ fibrils may dissociate into toxic Aβ protofilaments in the brain. Similar to the results of our study, alpha-synuclein fibrils, a major component of Lewy bodies that are a pathological hallmark of Parkinson’s disease, were disaggregated by interaction with noradrenaline and remodeled to the cytotoxic and insoluble alpha-synuclein oligomers [28]. Additionally, Li et al. demonstrated that catecholamine (L-DOPA) dissolved alpha-synuclein fibrils deposited in the mouse brain [12]. Given that DA is a neurotransmitter in the brain, and Aβ aggregates are deposited outside neural cells, they can interact with each other in the brain. Taken together, we hypothesize that the disaggregation process of Aβ fibrils is one of the possible causes of toxic Aβ protofibrils in the brain. Further experiments on the disaggregation of Aβ fibrils and generation of toxic Aβ protofibrils in the AD brain are required to test our hypothesis. Since an AD mouse model was already developed [29], it is possible to elucidate whether the disaggregation process of Aβ fibrils plays an important role in the pathological pathway to the onset or progression of AD. Our findings may help to further understand how Aβ exerts toxicity in the brains of patients with AD.

## 4. Materials and Methods

### 4.1. Chemicals

The human Aβ_1-42_ peptide (Cat. No. 4349-v) was purchased from the Peptide Institute, Inc (Osaka, Japan). EGCG was purchased from the Tokyo Chemical Industry Co., Ltd (Tokyo, Japan). DA hydrochloride was purchased from LKT Laboratories Inc (St. Paul, Minnesota, USA). All chemicals were of analytical-grade purity. The chemical solutions were freshly prepared in dimethyl sulfoxide (DMSO) and phosphate-buffered saline (PBS) buffer from lyophilized powder before each experiment.

### 4.2. Preparation of Aβ Fibrils and Disaggregated Aβ Solutions

Synthetic Aβ peptides were dissolved in hexafluoro-2-propanol for 10 min, and a 0.5 mM Aβ solution was then evaporated to dryness and stored at −30 °C until use. To produce Aβ fibril solutions, dried Aβ was resuspended in DMSO, followed by a brief vortexing and sonication for 1.5 min. The Aβ solvent was diluted in Ham’s F12 medium without phenol red (Research Institute for the Functional Peptides, Yamagata, Japan) to a concentration of 100 µM and incubated at 22 °C for a minimum of 90 h. For the disaggregation assay, Aβ fibril solutions (~90 µM) were incubated with the disaggregation reagents at different concentration ratios at 37 °C. Aliquots were collected at different time points from 0 to 24 h, flash-frozen in liquid nitrogen, and stored at −80 °C until further analysis.

### 4.3. ThT Fluorescence Measurement

Triplicates of 5 µL Aβ aliquots collected during disaggregation were mixed with 200 µL of 20 µM ThT in PBS. After pipetting them several times, the ThT fluorescence intensity was recorded with excitation at 450 nm and emission at 486 nm using a Varioskan Flash microplate reader (Thermo Fisher Scientific, Waltham, MA, USA).

### 4.4. MTS Cytotoxicity Assay

When we confirmed the cytotoxicity of the disaggregated Aβ, a 10-fold dilution of the disaggregated Aβ in the cell medium was required. Therefore, we needed a higher concentration of Aβ fibrils than the molar concentration determined and used in previously published papers (for example, 15 µM [11] and 22 µM [14]) to examine its cytotoxicity after disaggregation. The cytotoxicity of the Aβ samples was analyzed using the MTS assay (Promega, Madison, WI, USA), as previously described [30]. Briefly, the 90 µM Aβ solutions were preincubated in the absence or presence of the disaggregation reagents, and their corresponding solvent controls without Aβ were added to human neuroblastoma SH-SY5Y cells after a 10-fold dilution in a cell culture medium. After incubation for approximately 42 h at 37 °C, the supernatants from each well were carefully removed, and the MTS detection reagent was immediately added. We calculated the percentage of cell viability, with 100% representing the cells incubated in the solvent control: diluted DMSO with PBS.

### 4.5. Imaging by AFM

The Aβ samples were adsorbed onto a highly oriented pyrolytic graphite substrate (MikroMasch, Tallinn, Estonia) for 1 h and rinsed twice with MilliQ deionized water. Dry AFM imaging was performed in dynamic mode using a SPM-9700HT (Shimadzu Corporation, Kyoto, Japan) and silicon cantilevers (OMCL-AC200TS-RS, Olympus, Tokyo, Japan). For analysis of the height distribution, each AFM image (216 × 216 px) was flattened using Gwyddion 2.57 software, and the top 10% or 20% in height was taken as excerpts for generating the histogram.

### 4.6. CD Spectrometry Measurement

To evaluate the formation of the β-sheet structures derived from Aβ, CD spectral measurements were performed using a J-820 (JASCO, Tokyo, Japan). Before measurement, the solutions of every sample were exchanged with a 10 mM sodium phosphate buffer using Amicon ultra 0.5 mL, and 3 kDa (Merck Millipore, Darmstadt, Germany) to remove DMSO. Each spectrum of EGCG- or DA-treated Aβ samples was subtracted from the corresponding solvents without Aβ. Thermal stability was performed from 20–100 °C by increasing the temperature by 2 °C/min.
